# Evaluation of GPT-4 Accuracy in the Interpretation of Medical Imaging: Potential Benefits, Limitations, and the Future

**DOI:** 10.7759/cureus.87761

**Published:** 2025-07-12

**Authors:** Nikoloz Papiashvili, Christina Abshilava, Mohammad H Malik, Tinatin Dzindzibadze, Emeli J Anderson, Sopio Gagua, Vladimir Guruli, Kaveesha Amarasinghe, Nana Gonjilashvili, Irma Tchokhonelidze

**Affiliations:** 1 Faculty of Medicine, Tbilisi State Medical University, Tbilisi, GEO; 2 Department of Nephrology, Ingorokva High Medical Technology University Clinic, Tbilisi, GEO

**Keywords:** artificial intelligence, decision support systems, diagnostic imaging, generative pre-trained transformer, radiology

## Abstract

Introduction

The implementation of artificial intelligence (AI) in radiology as a medical decision support system has the potential to enhance diagnostic accuracy and improve patient outcomes. This retrospective study aimed to evaluate the diagnostic capabilities of GPT-4o in interpreting radiological imaging, specifically X-ray, CT, and MRI images, across various organ systems and disease types.

Methods

A total of 377 cases were collected and presented to GPT-4o with a standardized prompt and no clinical context. The responses were assessed by three independent raters using a five-point rating system.

Results

X-ray imaging exhibited a 2.21 times higher chance, on average, of being interpreted accurately compared to CT scans (odds ratio (OR): 2.21; 95% confidence interval (CI): 1.33 - 3.69), while pelvic imaging had a 6.25 times lower chance, on average, of being interpreted accurately when compared to images of the abdomen (OR: 0.16; 95% CI: 0.02 - 0.56). Additionally, neoplastic conditions had a 2.7 times lower chance, on average, of being interpreted accurately compared to bleeding conditions (OR: 0.37; 95% CI: 0.16 - 0.84).

Conclusion

A bimodal distribution of median ratings highlights an overreliance on comparability to prior image encounters and emphasizes the need to develop a systematic approach to image analysis. Future research should prioritize eliminating hallucination, establishing standardized evaluation criteria, and exploring methods to integrate visual and text-based data in a balanced manner. Additionally, efforts should be directed towards enhancing dataset diversity to improve the model's overall accuracy and generalizability.

## Introduction

The role of artificial intelligence (AI) in medicine has emerged as a significant area of research in recent years, and its capabilities have found applications in many aspects of healthcare. While both its text-based interpretation and image recognition skills are remarkable, the latter has been particularly impactful in areas such as diagnosing cancer on histopathology [1], differentiating between skin lesions [2], or assessing lung injury [3].

In the field of radiology, this technology has the potential to streamline the diagnostic workflow, enhance accuracy, improve patient outcomes by eliminating the need for additional, unnecessary procedures, and highlighting lesions that might otherwise be missed by human error [4]. This impact has led to a focus on the field, further amplified by the shortage of qualified radiologists in underserved communities and the technology’s ability to enable faster decision-making in emergency settings.

While existing research has explored the applications of AI in interpreting a variety of diagnostic modalities, organ systems, and conditions, the results have shown considerable variability [5-7]. Additionally, the interpretation of these results is often subjective, as the development of standardized assessment guidelines has proven to be challenging [8]. For this reason, many studies have used ChatGPT, which has demonstrated noteworthy diagnostic capabilities of its own [9-11], as a benchmark for comparison, even though its overall accuracy in this context remains difficult to ascertain.

This retrospective study aimed to establish a defined level of accuracy for GPT-4o’s capabilities in interpreting radiological images. To achieve this, 377 cases were collected, encompassing X-ray, CT, and MRI images, stratified by organ system and disease type. These were individually presented to the AI without clinical context and its responses were assessed by three independent raters, allowing us to evaluate its diagnostic performance and identify areas showing potential for implementation, as well as those requiring further improvement.

## Materials and methods

Data collection

Medical imagery was collected from Radiopaedia, an online peer-reviewed open-access radiology resource [12]. A target minimum of 300 cases was selected, considering the availability of applicable cases, as well as the sample sizes described by similar existing studies [5,13]. Our study included CT, MRI, and X-ray images of cases with a diagnostic certainty of “Certain” [14]. The cases were considered for inclusion randomly, but images with poor graphical quality and those with annotations, arrows, circles, or other edits were excluded. Due to technical limitations, only the thumbnail slice, which best depicts the findings and is selected by the case author during upload, was acquired from CT and MRI studies.

Each image was then reviewed by a consultant radiologist who either approved or rejected its inclusion based on whether the diagnosis could be suspected from the image alone, without clinical context. For all approved cases, we collected the file name, diagnosis, description, study modality, and downloaded the image. In the case of images lacking a description, one was provided by the consultant radiologist. Additionally, to ensure the proper attribution of all cases, the following details were collected separately: name of case contributor, radiopaedia identification number (rID), and digital object identifier (DOI) [15]. Case attributions for all cases can be found in the Appendix.

Data preparation

All images were checked to ensure they contained no identifying information. Duplicate cases were identified, with only one copy retained. All file names were replaced with numerical IDs to prevent the AI from obtaining additional information about the case. Each case was assigned a system category, which included abdomen, chest, extremities, head, pelvis, spine, and other, as well as one of the following type categories: bleeding, inflammatory, neoplastic, structural, normal, and other. The system category was selected based on the part of the body under study in the given image, while the type category was chosen based on the pathophysiology of the depicted condition. Bleeding conditions included hematomas, hemorrhagic stroke, and hemothorax, among others; inflammatory conditions included infectious diseases, autoimmune disorders, and inflammatory bowel disease; neoplastic conditions included benign or malignant tumors; structural conditions encompassed aneurysms, obstructions, hernias, fractures, tears, ulcers, congenital abnormalities, and more.

Prompt input and rating of responses

Each image was individually presented to GPT-4o, uploaded in .png or .jpg format, along with the following custom instructions: “You will be provided with an image depicting either an X-ray, CT scan, or MRI scan of the human body. Describe the findings shown in the image and name the most probable differential diagnoses based on the findings.” The description and differential diagnoses provided by GPT-4o were compared with the description and diagnosis of the case by three independent raters and scored using a five-point rating system. To ensure inter-rater reliability, the raters had no communication with one another and were not able to view others’ ratings.

The five-point rating system is as follows: 5=both the diagnosis and description of the image were accurate; 4=either the diagnosis or description of the image was accurate, but not both; 3=the diagnosis and description of the image were incorrect but another diagnosis was very similar; 2=the diagnosis and description of the image were incorrect but another diagnosis was possibly helpful in identifying the correct diagnosis; 1=the diagnosis and description of the image were incorrect and no diagnosis was possibly helpful in identifying the correct diagnosis. The median value of the three ratings was calculated for each case to be used during statistical analysis. In addition, a median score of 4 or 5 was defined as “Satisfactory”, while scores 1-3 were defined as “Poor”. This rating system was developed due to the lack of a standardized assessment guideline [8], with a similar approach as prior studies evaluating AI performance [9,10,13].

Statistical analysis

The analysis of the data was performed using R (version 4.3.1) (R Foundation, Vienna) [16]. A significance level of α=0.05 was assumed. The distributions of cases by modality, system, type, and rating are listed as frequencies and percentages. Pearson's chi-squared test and Fisher's exact test were used to evaluate the differences between ratings by modality, system, and type. Regression analysis was performed using a generalized linear model to assess the factors impacting the binary outcome (“Satisfactory” vs “Poor”). Adjusting for confounders was performed by stratifying the data during analysis. Fleiss’ κ (kappa) was calculated as a measure of inter-rater reliability between three independent raters.

## Results

In total, 377 cases were included in the study. Among modalities, CT scans comprised 192 (50.93%) cases, followed by X-ray and MRI images with 99 (26.26%) and 86 (22.81%) cases, respectively. By system, the abdomen, head, and chest had the highest representation, with 111 (29.44%), 88 (23.34%), and 80 (21.22%) cases, respectively. From the type categories, structural, neoplastic, and inflammatory conditions had the highest amount of cases, with 174 (46.15%), 78 (20.69%), and 62 (16.45%) cases, respectively. The exact distribution of cases by these characteristics is presented in Table 1. Fleiss’ kappa, a measure of inter-rater reliability, was calculated to be 0.717.

**Table 1:** Distribution of Cases Overall and by Modality

Characteristic	Total	CT	MRI	X-ray
	N=377	N=192 (n, %)	N=86 (n, %)	N=99 (n, %)
System				
Abdomen	111	83 (74.77%)	4 (3.6%)	24 (21.62%)
Chest	80	48 (60%)	1 (1.25%)	31 (38.75%)
Extremities	28	0 (0%)	11 (39.29%)	17 (60.71%)
Head	88	41 (46.59%)	45 (51.14%)	2 (2.27%)
Pelvis	31	16 (51.61%)	13 (41.94%)	2 (6.45%)
Spine	20	3 (15%)	12 (60%)	5 (25%)
Other	19	1 (5.26%)	0 (0%)	18 (94.74%)
Type				
Bleeding	35	33 (94.29%)	2 (5.71%)	0 (0%)
Inflammatory	62	31 (50%)	10 (16.13%)	21 (33.87%)
Neoplastic	78	37 (47.44%)	31 (39.74%)	10 (12.82%)
Structural	174	80 (45.98%)	38 (21.84%)	56 (32.18%)
Normal	5	0 (0%)	0 (0%)	5 (100%)
Other	23	11 (47.83%)	5 (21.74%)	7 (30.43%)

**Figure 1:**
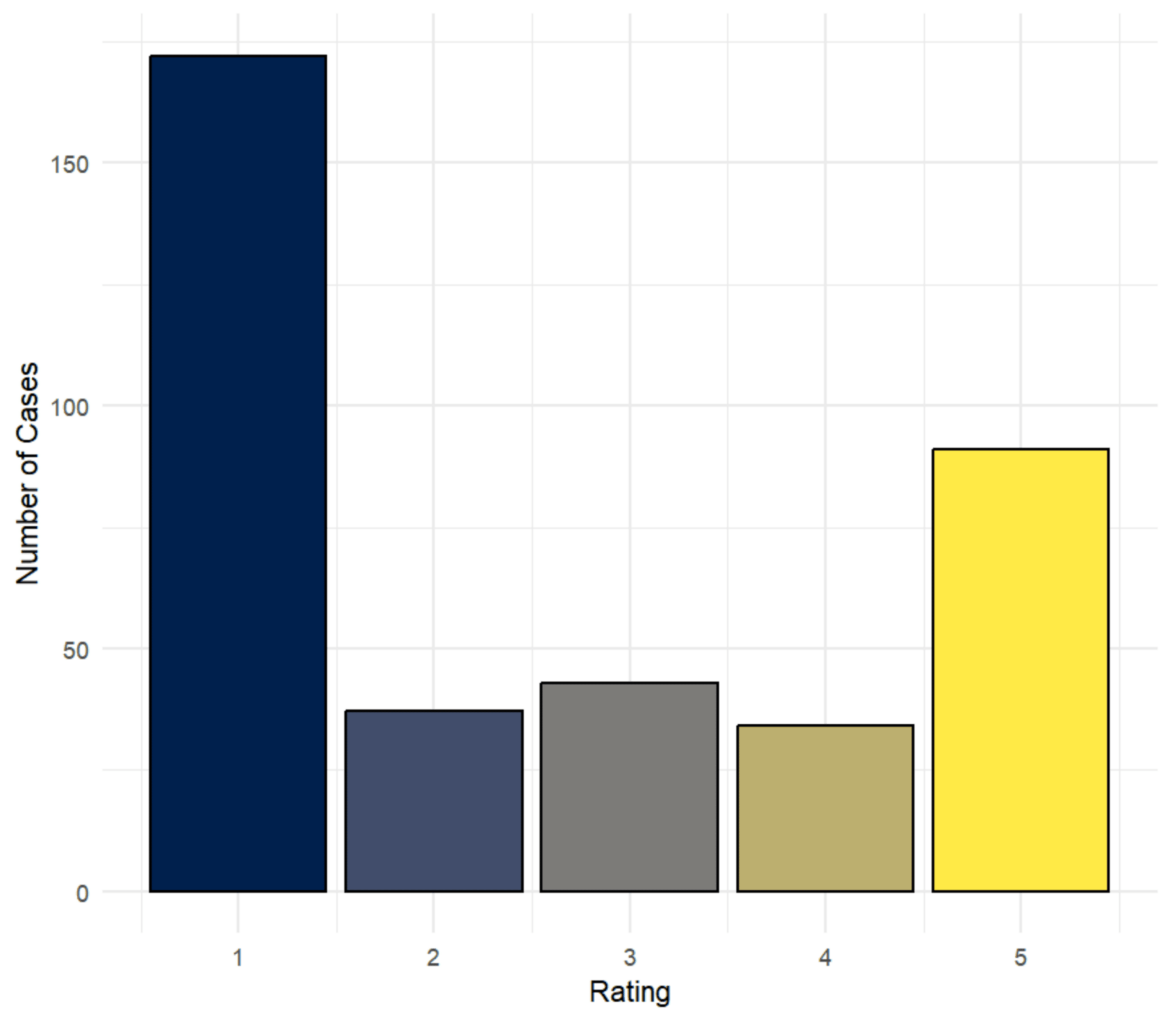
Distribution of Median Ratings for Responses Given by GPT-4o Across all Cases

The largest amount of cases received a median rating of 1 (n=172, 45.62%) or 5 (n=91, 24.14%) (Figure 1). When evaluating these results by modality, a statistically significant difference between the three categories was found for both the median score (p=0.034) and binary outcome (p=0.008). A detailed summary of these results is shown in Table 2. Additionally, when comparing cases by system, the difference was also found to be statistically significant for both the median score (p=0.001) and binary outcome (p<0.001). This distribution of cases is presented in Table 3. Stratification by type categories showed a statistically significant difference for the median score (p=0.018), but not for the binary outcome (p=0.2). An overview of these results can be found in Table 4.

**Table 2:** Median Ratings of Cases by Modality ** Pearson’s Chi-squared test.

Characteristic	CT	MRI	X-ray	Chi-square value	p-value^**^
	N=192 (n, %)	N=86 (n, %)	N=99 (n, %)		
Median				16.690	0.034
5	35 (18%)	22 (26%)	34 (34%)		
4	16 (8.3%)	8 (9.3%)	10 (10%)		
3	18 (9.4%)	12 (14%)	13 (13%)		
2	19 (9.9%)	11 (13%)	7 (7.1%)		
1	104 (54%)	33 (38%)	35 (35%)		
Rating				9.574	0.008
Satisfactory	51 (27%)	30 (35%)	44 (44%)		
Poor	141 (73%)	56 (65%)	55 (56%)		

**Table 3:** Median Ratings of Cases by System * n (%), ** Fisher's exact test, *** Pearson’s Chi-squared test.

Characteristic	Abdomen	Chest	Extremities	Head	Other	Pelvis	Spine	Chi-square value	p-value
	N=111^*^	N=80^*^	N=28^*^	N=88^*^	N=19^*^	N=31^*^	N=20^*^		
Median								N/A	0.001^**^
5	21 (19%)	13 (16%)	9 (32%)	29 (33%)	11 (58%)	2 (6.5%)	6 (30%)		
4	13 (12%)	8 (10%)	2 (7.1%)	6 (6.8%)	3 (16%)	0 (0%)	2 (10%)		
3	8 (7.2%)	8 (10%)	4 (14%)	14 (16%)	1 (5.3%)	5 (16%)	3 (15%)		
2	8 (7.2%)	9 (11%)	2 (7.1%)	11 (13%)	0 (0%)	5 (16%)	2 (10%)		
1	61 (55%)	42 (53%)	11 (39%)	28 (32%)	4 (21%)	19 (61%)	7 (35%)		
Rating								28.733	<0.001^***^
Satisfactory	34 (31%)	21 (26%)	11 (39%)	35 (40%)	14 (74%)	2 (6.5%)	8 (40%)		
Poor	77 (69%)	59 (74%)	17 (61%)	53 (60%)	5 (26%)	29 (94%)	12 (60%)		

**Table 4:** Median Ratings of Cases by Type * n (%), ** Fisher's exact test

Characteristic	Bleeding	Inflammatory	Neoplastic	Normal	Structural	Other	p-value^**^
	N=35^*^	N=62^*^	N=78^*^	N=5^*^	N=174^*^	N=23^*^	
Median							0.018
5	13 (37%)	10 (16%)	14 (18%)	2 (40%)	47 (27%)	5 (22%)	
4	4 (11%)	10 (16%)	6 (7.7%)	1 (20%)	9 (5.2%)	4 (17%)	
3	5 (14%)	4 (6.5%)	10 (13%)	1 (20%)	17 (9.8%)	6 (26%)	
2	4 (11%)	10 (16%)	7 (9.0%)	0 (0%)	15 (8.6%)	1 (4.3%)	
1	9 (26%)	28 (45%)	41 (53%)	1 (20%)	86 (49%)	7 (30%)	
Rating							0.2
Satisfactory	17 (49%)	20 (32%)	20 (26%)	3 (60%)	56 (32%)	9 (39%)	
Poor	18 (51%)	42 (68%)	58 (74%)	2 (40%)	118 (68%)	14 (61%)	

**Figure 2:**
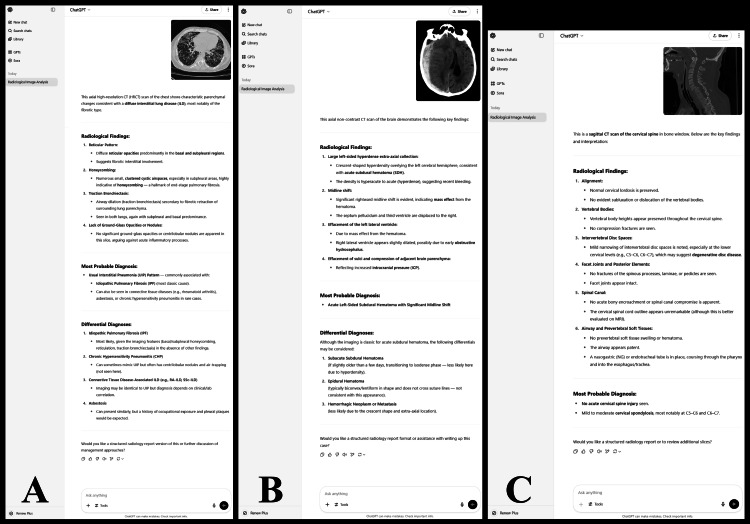
Examples of Responses Generated by GPT-4o (A) Case depicting usual interstitial pneumonia, identified correctly. (B) Case depicting acute subdural hemorrhage, identified correctly. (C) Case depicting a C6-C7 fracture dislocation, misidentified as normal.

A regression analysis assessed the relationship between modality, system, and type on the binary outcome (Table 5). Of these factors, the following were shown to be statistically significant: X-ray images had a 2.21 times higher chance on average to lead to a rating of 4 or 5 compared to CT scans (OR: 2.21; 95% CI: 1.33-3.69); images of the pelvis had a 6.25 times lower chance on average of leading to a score of 4 or 5 compared to images of the abdomen (OR: 0.16; 95% CI: 0.02-0.56); images in the “other” category of systems had a 6.34 times higher chance on average to lead to a rating of 4 or 5 compared to images of the abdomen (OR: 6.34; 95% CI: 2.23-20.92); images of neoplastic conditions had a 2.7 times lower chance on average of leading to a score of 4 or 5 compared to bleeding conditions (OR: 0.37; 95% CI: 0.16-0.84). Examples of responses given by GPT-4o are shown in Figure 2.

**Table 5:** Results of Regression Analysis * Statistically significant; Hyphen (-) indicates reference category.

Variables	OR (95% CI)
Modality	
CT	-
MRI	1.48 (0.85, 2.55)
X-ray^*^	2.21 (1.33, 3.69)
System	
Abdomen	-
Chest	0.81 (0.42, 1.52)
Extremities	1.47 (0.61, 3.44)
Head	1.50 (0.83, 2.70)
Pelvis^*^	0.16 (0.02, 0.56)
Spine	1.51 (0.55, 3.99)
Other^*^	6.34 (2.23, 20.92)
Type	
Bleeding	-
Inflammatory	0.50 (0.21, 1.18)
Neoplastic^*^	0.37 (0.16, 0.84)
Structural	0.50 (0.24, 1.05)
Normal	1.59 (0.24, 13.20)
Other	0.68 (0.23, 1.97)

## Discussion

The diagnostic capabilities of GPT-4o in radiology have been assessed in several specific applications, but few studies have quantitatively evaluated its performance across multiple imaging modalities and systems [7,17]. This study fills this gap by evaluating GPT-4o’s performance with a diverse dataset, identifying strengths, pitfalls, and avenues requiring further improvement. Our results highlighted factors significantly affecting diagnostic accuracy, such as pelvic, neoplastic, and X-ray images. They also provide insight into the limitations affecting the overall accuracy of this technology and areas of interest for future development.

Several noteworthy findings emerged from the results. Statistically significant differences were observed in the scores of responses categorized by modality, system, and type. To assess these findings in more detail, regression analysis was performed, which showing that, on average, GPT-4o produced more favorable outcomes when working with X-ray images (OR: 2.21, 95% CI: 1.33-3.69). A similar finding was observed with the "other" category of systems (OR: 6.34; 95% CI: 2.23-20.92). The opposite result was seen with imaging of the pelvis (OR: 0.16; 95% CI: 0.02-0.56) and neoplastic conditions (OR: 0.37; 95% CI: 0.16-0.84), indicating that GPT-4o struggles considerably when evaluating these kinds of images. Additionally, inter-rater reliability was assessed, yielding a Fleiss' kappa of 0.717, indicating substantial agreement between the three independent raters.

Among X-ray images, 44% of cases achieved a "Satisfactory" rating, comparable to the 40.50% and 47% with an "Acceptable" rating in a study involving chest X-rays by Lee et al. [9]. Additionally, the increased success rate with this modality over CT scans could be attributed to the greater availability of X-ray images, which were likely more commonly presented to the AI, enabling improvement over time. Despite generally better performance with X-rays, it should be noted that almost all cases with bleeding conditions were CT scans. GPT-4o achieved a satisfactory rating in 49% of these cases, a higher percentage than most other case types. Furthermore, regression analysis indicated worse outcomes for most condition types, even though not all results reached statistical significance.

Regarding pelvic imaging, GPT-4o would often admit to being unable to identify whether the image depicted female or male pelvic anatomy, suggesting separate differential diagnoses based on either option. Other times, it would mistake an enlarged prostate or a distended bladder for the uterus, completely missing the diagnosis. These findings underline the critical need for further training in the interpretation of pelvic imaging, specifically in differentiating between normal male and female pelvic anatomy, before abnormal imaging can be introduced. Responses from the "other" category of systems achieved a satisfactory rating in 74% of cases, substantiated by the findings in regression analysis (OR: 6.34; 95% CI: 2.23-20.92). Although this group of cases consisted of a variety of conditions, a significant number of esophageal X-rays were included, suggesting the need for a more detailed evaluation of this subset of cases.

The overall distribution of median ratings demonstrates a bimodal distribution, indicating that most cases were interpreted either completely incorrectly or entirely without error, with only a small percentage of responses being partially accurate. This finding suggests that the AI adopts an "all or nothing" approach, expertly recognizing anything it has seen before but being practically unable to work with anything it has not visually encountered, despite having access to theoretical knowledge on these conditions. Another important factor negatively impacting accuracy was hallucinations, which are a commonly encountered, well-documented limitation of ChatGPT, representing false or misleading statements provided by an AI, presented as fact [9,17,18]. As diagnostic validity is of the utmost importance in radiology, the presence of this phenomenon underscores the importance of eliminating errors like these before the AI model can be reliably implemented in clinical settings.

Moreover, the study purposefully withheld key information, such as text-based descriptions and clinical history, which are typically available to human radiologists. This was done to eliminate potential confounding factors, both of which have been shown to significantly influence the responses provided by the AI. A study by Wu et al. found that the accuracy of responses significantly improved with the inclusion of clinical history alongside the image, relying heavily on details like the patient's past medical history [17]. Buckley et al. evaluated this phenomenon further by adding misleading text to medical images, such as a suggestion from a fictional colleague [19]. Their results showed that the AI would prioritize text-based information over the findings seen in the image, missing diagnoses even when the image alone would otherwise have been accurately interpreted.

While the precise mechanisms behind the AI’s decision-making process remain unclear even to the most seasoned researchers, these findings offer valuable insight into certain characteristics. The bimodal distribution of median ratings strongly suggests that the model relies primarily on comparing the prompted image to one it has encountered prior, rather than analyzing the various components of the image in detail. This could also explain the recurring hallucinations, which are likely a result of the model making incorrect comparisons. Furthermore, since the model is able to process text-based data with greater accuracy [20], it relies too heavily on clinical history and case descriptions, when they are available.

Future research should focus on methods to combat these pitfalls. One possibility may be to instruct the AI to analyze the image in sections, with each part contributing to a consolidated list of potential diagnoses. This approach has shown particular success in the field of pathology [1]. Additionally, text-based data could be incorporated as a secondary source of information, supporting or challenging certain diagnoses that the model has already considered.

Multiple limitations were encountered during this study, which should be considered during the interpretation of results. The inability to provide more than one slice for each case was an important technical limitation, as it would particularly impact the interpretation of MRI and CT scans, omitting valuable information that AI might have derived from additional slices. This provides a potential explanation for the results observed in the regression analysis regarding X-ray images. Furthermore, the dataset was not evenly distributed, with 192 cases being CT scans and only 86 and 99 cases representing MRI and X-ray images, respectively. Some medical conditions were represented multiple times, while others appeared only once. These factors could be considered a type of selection bias, caused by technical limitations and lack of availability of a more diverse dataset. Future work should focus on the development of an extensive and diverse dataset, as this is essential for enhancing the model's accuracy and generalizability.

Another concern encountered in many AI studies stems from the lack of an objective, standardized assessment guidelines, with each study developing a unique approach to evaluating responses. In our case, we opted for the five-point rating system, following the approach of prior research in the field. However, in order to avoid this measurement bias, future work should prioritize the development of clear, standardized evaluation criteria to ensure consistency and comparability across studies.

## Conclusions

The objective of the study was to evaluate the diagnostic capabilities of GPT-4o in radiological imaging, taking into account the factors influencing accuracy, limitations, and potential areas for improvement. The results demonstrated higher accuracy with X-ray images, while pelvic imaging and neoplastic conditions posed notable challenges. A bimodal distribution in the quality of responses suggests that the AI relies heavily on prior encounters with similar images, limiting generalizability. Furthermore, hallucination was identified as a significant concern for the safe integration of GPT-4o into medical settings, highlighting the need for strategies to mitigate this issue.

Avenues for future research should include the creation of standardized assessment guidelines, the development of an extensive dataset, and the implementation of a systematic approach to image analysis. In addition, exploring methods to incorporate text-based data by having it complement rather than override the image will be essential in improving the model’s overall diagnostic capabilities.

## Appendices

**Table 6:** Attributions for All Cases Collected from Radiopaedia

ID	Case	Attribution
1	Abdominal aortic aneurysm	Case courtesy of The Radswiki, Radiopaedia.org, rID: 11151, https://doi.org/10.53347/rID-11151
2	Abdominal aortic aneurysm	Case courtesy of Ahmed Abdrabou, Radiopaedia.org, rID: 25855, https://doi.org/10.53347/rID-25855
3	Abdominal aortic aneurysm	Case courtesy of Sarita Bishnoi, Radiopaedia.org, rID: 25985, https://doi.org/10.53347/rID-25985
4	Abdominal aortic aneurysm	Case courtesy of David Cuete, Radiopaedia.org, rID: 29248, https://doi.org/10.53347/rID-29248
5	Abdominal aortic aneurysm	Case courtesy of Mohamed Hossam Shebl, Radiopaedia.org, rID: 20461, https://doi.org/10.53347/rID-20461
6	Abdominal ectopic pregnancy in the second trimester	Case courtesy of Dr. Debraj Saha, Radiopaedia.org, rID: 198570, https://doi.org/10.53347/rID-198570
7	Abdominal wall and retroperitoneal tuberculosis	Case courtesy of Michael P. Hartung, Radiopaedia.org, rID: 88137, https://doi.org/10.53347/rID-88137
8	Abdominal wall haematoma	Case courtesy of Jeremy Jones, Radiopaedia.org, rID: 8082, https://doi.org/10.53347/rID-8082
9	Abernethy malformation type 1b - acute spontaneous hepatic haemorrhage	Case courtesy of Liz Silverstone, Radiopaedia.org, rID: 92752, https://doi.org/10.53347/rID-92752
10	Achalasia	Case courtesy of Jan Frank Gerstenmaier, Radiopaedia.org, rID: 24937, https://doi.org/10.53347/rID-24937
11	Achalasia	Case courtesy of Mohammad Taghi Niknejad, Radiopaedia.org, rID: 60393, https://doi.org/10.53347/rID-60393
12	Achalasia	Case courtesy of Mohammad Taghi Niknejad, Radiopaedia.org, rID: 89229, https://doi.org/10.53347/rID-89229
13	Achalasia	Case courtesy of Bahman Rasuli, Radiopaedia.org, rID: 93396, https://doi.org/10.53347/rID-93396
14	Achalasia	Case courtesy of Frank Gaillard, Radiopaedia.org, rID: 14538, https://doi.org/10.53347/rID-14538
15	Achalasia	Case courtesy of Mohammad Taghi Niknejad, Radiopaedia.org, rID: 100002, https://doi.org/10.53347/rID-100002
16	Achalasia	Case courtesy of Mohammad Taghi Niknejad, Radiopaedia.org, rID: 150032, https://doi.org/10.53347/rID-150032
17	Achalasia	Case courtesy of Mohammad Taghi Niknejad, Radiopaedia.org, rID: 157757, https://doi.org/10.53347/rID-157757
18	Achalasia	Case courtesy of Mohammad Taghi Niknejad, Radiopaedia.org, rID: 96436, https://doi.org/10.53347/rID-96436
19	Achalasia	Case courtesy of Mohammad Taghi Niknejad, Radiopaedia.org, rID: 95716, https://doi.org/10.53347/rID-95716
20	Achilles tendon ossification	Case courtesy of Domenico Nicoletti, Radiopaedia.org, rID: 46824, https://doi.org/10.53347/rID-46824
21	Achilles tendon ossification	Case courtesy of M Weber, Radiopaedia.org, rID: 5587, https://doi.org/10.53347/rID-5587
22	Achilles tendon ossification	Case courtesy of Roberto Schubert, Radiopaedia.org, rID: 18474, https://doi.org/10.53347/rID-18474
23	Achilles tendon tear	Case courtesy of Frank Gaillard, Radiopaedia.org, rID: 2572, https://doi.org/10.53347/rID-2572
24	Achilles tendon tear	Case courtesy of Arthur Daire, Radiopaedia.org, rID: 31266, https://doi.org/10.53347/rID-31266
25	Acromial fracture	Case courtesy of Bahman Rasuli, Radiopaedia.org, rID: 72090, https://doi.org/10.53347/rID-72090
26	Acromioclavicular arthritis - rotator cuff tear	Case courtesy of Paresh K Desai, Radiopaedia.org, rID: 10890, https://doi.org/10.53347/rID-10890
27	Acromioclavicular joint injury	Case courtesy of Frank Gaillard, Radiopaedia.org, rID: 5842, https://doi.org/10.53347/rID-5842
28	Acromioclavicular joint injury	Case courtesy of Frank Gaillard, Radiopaedia.org, rID: 35984, https://doi.org/10.53347/rID-35984
29	Acute disseminated encephalomyelitis	Case courtesy of Frank Gaillard, Radiopaedia.org, rID: 37704, https://doi.org/10.53347/rID-37704
30	Acute mesenteric haematoma with active bleeding	Case courtesy of Maxime St-Amant, Radiopaedia.org, rID: 20112, https://doi.org/10.53347/rID-20112
31	Acute renal artery occlusion	Case courtesy of Dr Ngo Tuan Minh, Radiopaedia.org, rID: 86586, https://doi.org/10.53347/rID-86586
32	Adenomyomatosis of the gallbladder	Case courtesy of Erik Ranschaert, Radiopaedia.org, rID: 12142, https://doi.org/10.53347/rID-12142
33	Adhesional small bowel obstruction	Case courtesy of Ian Bickle, Radiopaedia.org, rID: 48397, https://doi.org/10.53347/rID-48397
34	Adhesional small bowel obstruction	Case courtesy of Mohammad Salem Amer, Radiopaedia.org, rID: 184647, https://doi.org/10.53347/rID-184647
35	Ankylosing spondylitis	Case courtesy of RMH Core Conditions, Radiopaedia.org, rID: 33844, https://doi.org/10.53347/rID-33844
36	Anterior abdominal wall hernia with bowel obstruction	Case courtesy of Ammar Ashraf, Radiopaedia.org, rID: 171221, https://doi.org/10.53347/rID-171221
37	Anterior calcaneal process fracture	Case courtesy of Magdalena Chmiel-Nowak, Radiopaedia.org, rID: 74654, https://doi.org/10.53347/rID-74654
38	Aortic dissection with rupture into pericardium	Case courtesy of Frank Gaillard, Radiopaedia.org, rID: 12384, https://doi.org/10.53347/rID-12384
39	Aortic dissection with rupture into the pericardial sac	Case courtesy of J. Ray Ballinger, Radiopaedia.org, rID: 23726, https://doi.org/10.53347/rID-23726
40	Aortic stent	Case courtesy of Ian Bickle, Radiopaedia.org, rID: 33202, https://doi.org/10.53347/rID-33202
41	Aortic valve endocarditis	Case courtesy of Matthew Tse, Radiopaedia.org, rID: 87209, https://doi.org/10.53347/rID-87209
42	Apical non-small cell lung cancer	Case courtesy of Frank Gaillard, Radiopaedia.org, rID: 8570, https://doi.org/10.53347/rID-8570
43	Astrocytoma	Case courtesy of Roberto Schubert, Radiopaedia.org, rID: 16279, https://doi.org/10.53347/rID-16279
44	Atlanto-occipital dissociation	Case courtesy of Andrew Dixon, Radiopaedia.org, rID: 45395, https://doi.org/10.53347/rID-45395
45	Atrial myxoma	Case courtesy of Frank Gaillard, Radiopaedia.org, rID: 8544, https://doi.org/10.53347/rID-8544
46	Autosomal dominant polycystic kidney disease	Case courtesy of Frank Gaillard, Radiopaedia.org, rID: 5203, https://doi.org/10.53347/rID-5203
47	Baker cyst - ruptured	Case courtesy of Marina Goymer, Radiopaedia.org, rID: 25233, https://doi.org/10.53347/rID-25233
48	Baker cyst with loose intracystic bodies	Case courtesy of Roberto Schubert, Radiopaedia.org, rID: 18675, https://doi.org/10.53347/rID-18675
49	Barrett oesophagus	Case courtesy of Matt A. Morgan, Radiopaedia.org, rID: 44421, https://doi.org/10.53347/rID-44421
50	Basal cell adenoma - parotid gland	Case courtesy of Fateme Hosseinabadi, Radiopaedia.org, rID: 69973, https://doi.org/10.53347/rID-69973
51	Basal cell carcinoma - external auditory canal	Case courtesy of Bahman Rasuli, Radiopaedia.org, rID: 78944, https://doi.org/10.53347/rID-78944
52	Basal ganglia haemorrhage	Case courtesy of Frank Gaillard, Radiopaedia.org, rID: 2764, https://doi.org/10.53347/rID-2764
53	Basal ganglia haemorrhage	Case courtesy of Mark Rodrigues, Radiopaedia.org, rID: 58762, https://doi.org/10.53347/rID-58762
54	Basal ganglia haemorrhage	Case courtesy of Frank Gaillard, Radiopaedia.org, rID: 2672, https://doi.org/10.53347/rID-2672
55	Basal ganglia haemorrhage	Case courtesy of Frank Gaillard, Radiopaedia.org, rID: 10598, https://doi.org/10.53347/rID-10598
56	Basilar artery aneurysm	Case courtesy of Lee Condon, Radiopaedia.org, rID: 56261, https://doi.org/10.53347/rID-56261
57	Basilar artery stenosis	Case courtesy of Bruno Di Muzio, Radiopaedia.org, rID: 30288, https://doi.org/10.53347/rID-30288
58	Basilar tip aneurysm	Case courtesy of Ian Bickle, Radiopaedia.org, rID: 77655, https://doi.org/10.53347/rID-77655
59	Basilar tip aneurysm	Case courtesy of Matt A. Morgan, Radiopaedia.org, rID: 53912, https://doi.org/10.53347/rID-53912
60	Benign metastasising leiomyoma	Case courtesy of Paul Leong, Radiopaedia.org, rID: 26803, https://doi.org/10.53347/rID-26803
61	Benign prostatic hyperplasia	Case courtesy of Frank Gaillard, Radiopaedia.org, rID: 2586, https://doi.org/10.53347/rID-2586
62	Bilateral atelectasis	Case courtesy of Andrew Murphy, Radiopaedia.org, rID: 48666, https://doi.org/10.53347/rID-48666
63	Bilateral hydronephrosis	Case courtesy of Jeremy Jones, Radiopaedia.org, rID: 6222, https://doi.org/10.53347/rID-6222
64	Bilateral ovarian fibroma	Case courtesy of Domenico Nicoletti, Radiopaedia.org, rID: 44568, https://doi.org/10.53347/rID-44568
65	Biliary cystadenoma	Case courtesy of The Radswiki, Radiopaedia.org, rID: 11234, https://doi.org/10.53347/rID-11234
66	Biliary cystadenoma	Case courtesy of Ammar Ashraf, Radiopaedia.org, rID: 91658, https://doi.org/10.53347/rID-91658
67	Bleeding peptic ulcer	Case courtesy of Henry Knipe, Radiopaedia.org, rID: 42912, https://doi.org/10.53347/rID-42912
68	Boerhaave syndrome	Case courtesy of Brendon Friesen, Radiopaedia.org, rID: 28804, https://doi.org/10.53347/rID-28804
69	Boerhaave syndrome	Case courtesy of Ahmed Abdrabou, Radiopaedia.org, rID: 28895, https://doi.org/10.53347/rID-28895
70	Boerhaave syndrome	Case courtesy of Craig Hacking, Radiopaedia.org, rID: 39382, https://doi.org/10.53347/rID-39382
71	Bronchiectasis	Case courtesy of The Radswiki, Radiopaedia.org, rID: 11258, https://doi.org/10.53347/rID-11258
72	Bronchiectasis	Case courtesy of Frank Gaillard, Radiopaedia.org, rID: 2587, https://doi.org/10.53347/rID-2587
73	Bronchiolitis obliterans	Case courtesy of Tee Yu Jin, Radiopaedia.org, rID: 71538, https://doi.org/10.53347/rID-71538
74	Burst fracture	Case courtesy of Ahmed Abdrabou, Radiopaedia.org, rID: 22980, https://doi.org/10.53347/rID-22980
75	Burst fracture	Case courtesy of Frank Gaillard, Radiopaedia.org, rID: 4635, https://doi.org/10.53347/rID-4635
76	Burst fracture with cauda equina compression	Case courtesy of Roberto Schubert, Radiopaedia.org, rID: 17537, https://doi.org/10.53347/rID-17537
77	Cannonball lung metastasis	Case courtesy of Sarah AlJahdali, Radiopaedia.org, rID: 77634, https://doi.org/10.53347/rID-77634
78	Carbon monoxide poisoning	Case courtesy of Hidayatullah Hamidi, Radiopaedia.org, rID: 65524, https://doi.org/10.53347/rID-65524
79	Cauda equina neuroendocrine tumour	Case courtesy of Frank Gaillard, Radiopaedia.org, rID: 19546, https://doi.org/10.53347/rID-19546
80	Cauda equina neuroendocrine tumour	Case courtesy of Frank Gaillard, Radiopaedia.org, rID: 157299, https://doi.org/10.53347/rID-157299
81	Cavernous haemangioma	Case courtesy of Frank Gaillard, Radiopaedia.org, rID: 9773, https://doi.org/10.53347/rID-9773
82	Cavernous haemangioma	Case courtesy of Frank Gaillard, Radiopaedia.org, rID: 9972, https://doi.org/10.53347/rID-9972
83	Cavernous haemangioma	Case courtesy of Frank Gaillard, Radiopaedia.org, rID: 34054, https://doi.org/10.53347/rID-34054
84	Cavernous haemangioma	Case courtesy of Ahmed Abdrabou, Radiopaedia.org, rID: 44301, https://doi.org/10.53347/rID-44301
85	Cerebellar agenesis with pontocerebellar hypoplasia	Case courtesy of Ernest Lekgabe, Radiopaedia.org, rID: 57224, https://doi.org/10.53347/rID-57224
86	Cerebellar atrophy	Case courtesy of Ahmed Almuslim, Radiopaedia.org, rID: 6807, https://doi.org/10.53347/rID-6807
87	Cerebellar haemorrhage	Case courtesy of David Cuete, Radiopaedia.org, rID: 27193, https://doi.org/10.53347/rID-27193
88	Cerebellar infarct with upward transtentorial herniation	Case courtesy of Frank Gaillard, Radiopaedia.org, rID: 9976, https://doi.org/10.53347/rID-9976
89	Cerebellopontine angle schwannoma	Case courtesy of Adam Eid Ramsey, MD, Radiopaedia.org, rID: 71378, https://doi.org/10.53347/rID-71378
90	Cerebral abscess	Case courtesy of Frank Gaillard, Radiopaedia.org, rID: 5150, https://doi.org/10.53347/rID-5150
91	Cerebral amyloid angiopathy and lobar haemorrhage	Case courtesy of RMH Neuropathology, Radiopaedia.org, rID: 24877, https://doi.org/10.53347/rID-24877
92	Cerebral infarction	Case courtesy of Bruno Di Muzio, Radiopaedia.org, rID: 49837, https://doi.org/10.53347/rID-49837
93	Cerebral vein thrombosis	Case courtesy of Frank Gaillard, Radiopaedia.org, rID: 4408, https://doi.org/10.53347/rID-4408
94	Cerebral venous sinus thrombosis	Case courtesy of Nikos Karapasias, Radiopaedia.org, rID: 25388, https://doi.org/10.53347/rID-25388
95	Cervical fracture dislocation C5-C6	Case courtesy of Samir Benoudina, Radiopaedia.org, rID: 47581, https://doi.org/10.53347/rID-47581
96	Cervical fracture dislocation C6-C7	Case courtesy of Frank Gaillard, Radiopaedia.org, rID: 4021, https://doi.org/10.53347/rID-4021
97	Cholelithiasis	Case courtesy of Behrang Amini, Radiopaedia.org, rID: 35953, https://doi.org/10.53347/rID-35953
98	Chronic eosinophilic pneumonia	Case courtesy of Henry Knipe, Radiopaedia.org, rID: 39331, https://doi.org/10.53347/rID-39331
99	Cytomegalovirus (CMV) ventriculitis and encephalitis	Case courtesy of Frank Gaillard, Radiopaedia.org, rID: 5416, https://doi.org/10.53347/rID-5416
100	Colon cancer with duodenal invasion	Case courtesy of Roberto Schubert, Radiopaedia.org, rID: 16278, https://doi.org/10.53347/rID-16278
101	Colonic diverticula	Case courtesy of Jeremy Jones, Radiopaedia.org, rID: 6200, https://doi.org/10.53347/rID-6200
102	Creutzfeldt-Jakob disease	Case courtesy of Ryan Thibodeau, Radiopaedia.org, rID: 166711, https://doi.org/10.53347/rID-166711
103	Cystic bronchiectasis	Case courtesy of Bruno Di Muzio, Radiopaedia.org, rID: 18289, https://doi.org/10.53347/rID-18289
104	Cystic papillary renal cell carcinoma	Case courtesy of Roberto Schubert, Radiopaedia.org, rID: 15780, https://doi.org/10.53347/rID-15780
105	Dacryocystitis	Case courtesy of Travis Fahrenhorst-Jones, Radiopaedia.org, rID: 91912, https://doi.org/10.53347/rID-91912
106	Dandy-Walker malformation	Case courtesy of Frank Gaillard, Radiopaedia.org, rID: 4593, https://doi.org/10.53347/rID-4593
107	Dandy-Walker malformation	Case courtesy of Frank Gaillard, Radiopaedia.org, rID: 15808, https://doi.org/10.53347/rID-15808
108	Dengue encephalitis	Case courtesy of Utkarsh Kabra, Radiopaedia.org, rID: 151882, https://doi.org/10.53347/rID-151882
109	Dens fracture and epidural haematoma	Case courtesy of Andrew Dixon, Radiopaedia.org, rID: 31764, https://doi.org/10.53347/rID-31764
110	Depressed skull fracture	Case courtesy of Ian Bickle, Radiopaedia.org, rID: 29473, https://doi.org/10.53347/rID-29473
111	Depressed skull fracture	Case courtesy of Hani M. Al Salam, Radiopaedia.org, rID: 13084, https://doi.org/10.53347/rID-13084
112	Descending necrotising mediastinitis	Case courtesy of David Clemo Steel, Radiopaedia.org, rID: 47785, https://doi.org/10.53347/rID-47785
113	Diffuse alveolar haemorrhage	Case courtesy of Bruno Di Muzio, Radiopaedia.org, rID: 61650, https://doi.org/10.53347/rID-61650
114	Diffuse axonal injury	Case courtesy of Magdalena Chmiel-Nowak, Radiopaedia.org, rID: 75309, https://doi.org/10.53347/rID-75309
115	Diffuse axonal injury	Case courtesy of Ahmed Abdrabou, Radiopaedia.org, rID: 67569, https://doi.org/10.53347/rID-67569
116	Diffuse cerebellar atrophy	Case courtesy of Fakhry Mahmoud Ebouda, Radiopaedia.org, rID: 151978, https://doi.org/10.53347/rID-151978
117	Diverticular disease	Case courtesy of Jeremy Jones, Radiopaedia.org, rID: 6197, https://doi.org/10.53347/rID-6197
118	Dropped gallstones	Case courtesy of Chris O'Donnell, Radiopaedia.org, rID: 47256, https://doi.org/10.53347/rID-47256
119	Duodenal diverticulitis	Case courtesy of Derek Smith, Radiopaedia.org, rID: 92475, https://doi.org/10.53347/rID-92475
120	Duodenal diverticulum	Case courtesy of The Radswiki, Radiopaedia.org, rID: 11376, https://doi.org/10.53347/rID-11376
121	Duodenal diverticulum	Case courtesy of Frank Gaillard, Radiopaedia.org, rID: 14539, https://doi.org/10.53347/rID-14539
122	Duodenojejunal enteritis	Case courtesy of Ian Bickle, Radiopaedia.org, rID: 47153, https://doi.org/10.53347/rID-47153
123	Ebstein anomaly	Case courtesy of Maxime St-Amant, Radiopaedia.org, rID: 20700, https://doi.org/10.53347/rID-20700
124	Emphysematous pyelonephritis	Case courtesy of Ahmed Abdrabou, Radiopaedia.org, rID: 27191, https://doi.org/10.53347/rID-27191
125	Endometriosis of the urinary bladder	Case courtesy of Natalie Yang, Radiopaedia.org, rID: 6880, https://doi.org/10.53347/rID-6880
126	End-stage rheumatoid arthritis of the shoulder	Case courtesy of Domenico Nicoletti, Radiopaedia.org, rID: 180740, https://doi.org/10.53347/rID-180740
127	Eosinophilic granulomatosis with polyangiitis	Case courtesy of Yaïr Glick, Radiopaedia.org, rID: 70246, https://doi.org/10.53347/rID-70246
128	Epidural haematoma	Case courtesy of David Cuete, Radiopaedia.org, rID: 29440, https://doi.org/10.53347/rID-29440
129	Epiglottitis	Case courtesy of Maxime St-Amant, Radiopaedia.org, rID: 26840, https://doi.org/10.53347/rID-26840
130	Epiphrenic diverticulum	Case courtesy of Alexandra Stanislavsky, Radiopaedia.org, rID: 20257, https://doi.org/10.53347/rID-20257
131	Epithelioid mesothelioma	Case courtesy of Bruno Di Muzio, Radiopaedia.org, rID: 75229, https://doi.org/10.53347/rID-75229
132	Extensive abdominopelvic hydatidosis	Case courtesy of Michael P. Hartung, Radiopaedia.org, rID: 83238, https://doi.org/10.53347/rID-83238
133	Extradural haematoma	Case courtesy of Ian Bickle, Radiopaedia.org, rID: 30036, https://doi.org/10.53347/rID-30036
134	Extradural hematoma, subdural hematomas, cerebral contusions and subgaleal hematoma	Case courtesy of Frank Gaillard, Radiopaedia.org, rID: 19252, https://doi.org/10.53347/rID-19252
135	Finger proximal interphalangeal (PIP) joint septic arthritis and osteomyelitis	Case courtesy of Craig Hacking, Radiopaedia.org, rID: 81699, https://doi.org/10.53347/rID-81699
136	Follicular bronchiolitis	Case courtesy of Akos Jaray, Radiopaedia.org, rID: 75175, https://doi.org/10.53347/rID-75175
137	Frontal sinus fracture	Case courtesy of David Cuete, Radiopaedia.org, rID: 26072, https://doi.org/10.53347/rID-26072
138	Full-thickness external rectal prolapse	Case courtesy of Vikas Shah, Radiopaedia.org, rID: 50428, https://doi.org/10.53347/rID-50428
139	Gallbladder carcinoma	Case courtesy of Ahmed Abdrabou, Radiopaedia.org, rID: 33025, https://doi.org/10.53347/rID-33025
140	Gallbladder empyema	Case courtesy of Ian Bickle, Radiopaedia.org, rID: 21751, https://doi.org/10.53347/rID-21751
141	Gallbladder empyema	Case courtesy of Ian Bickle, Radiopaedia.org, rID: 24329, https://doi.org/10.53347/rID-24329
142	Gangrenous cholecystitis	Case courtesy of Frank Gaillard, Radiopaedia.org, rID: 19134, https://doi.org/10.53347/rID-19134
143	Gastric diverticulum	Case courtesy of Mohammad Taghi Niknejad, Radiopaedia.org, rID: 151407, https://doi.org/10.53347/rID-151407
144	Gastric outlet obstruction - gastric adenocarcinoma	Case courtesy of Ian Bickle, Radiopaedia.org, rID: 59301, https://doi.org/10.53347/rID-59301
145	Gastric ulcers	Case courtesy of Michael P. Hartung, Radiopaedia.org, rID: 65709, https://doi.org/10.53347/rID-65709
146	Giant coronary artery aneurysms	Case courtesy of David Cuevas, Radiopaedia.org, rID: 67499, https://doi.org/10.53347/rID-67499
147	Giant serpentine middle cerebral artery aneurysm	Case courtesy of Peter Mitchell, Radiopaedia.org, rID: 34350, https://doi.org/10.53347/rID-34350
148	Giant urinary bladder diverticulum	Case courtesy of Ashesh Ishwarlal Ranchod, Radiopaedia.org, rID: 167582, https://doi.org/10.53347/rID-167582
149	Glioblastoma	Case courtesy of Frank Gaillard, Radiopaedia.org, rID: 5292, https://doi.org/10.53347/rID-5292
150	Gunshot injury	Case courtesy of Alexandra Stanislavsky, Radiopaedia.org, rID: 13204, https://doi.org/10.53347/rID-13204
151	Haemoperitoneum post-traumatic splenic injury	Case courtesy of Jeremy Jones, Radiopaedia.org, rID: 14361, https://doi.org/10.53347/rID-14361
152	Haemopneumothorax	Case courtesy of Andrew Van, Radiopaedia.org, rID: 44657, https://doi.org/10.53347/rID-44657
153	Haemorrhagic cerebral infarction	Case courtesy of Ahmed Abdrabou, Radiopaedia.org, rID: 25281, https://doi.org/10.53347/rID-25281
154	Haemothorax	Case courtesy of Andrew Dixon, Radiopaedia.org, rID: 31522, https://doi.org/10.53347/rID-31522
155	Hepatic congestion	Case courtesy of Heba Abdelmonem, Radiopaedia.org, rID: 63799, https://doi.org/10.53347/rID-63799
156	Hepatic haemangioendothelioma	Case courtesy of Amr Farouk, Radiopaedia.org, rID: 44487, https://doi.org/10.53347/rID-44487
157	Hepatic steatosis	Case courtesy of Chris O'Donnell, Radiopaedia.org, rID: 27910, https://doi.org/10.53347/rID-27910
158	Hepatocellular carcinoma	Case courtesy of Suraindra Mark Rajadurai, Radiopaedia.org, rID: 62295, https://doi.org/10.53347/rID-62295
159	Herpes simplex virus encephalitis	Case courtesy of Amit Chacko, Radiopaedia.org, rID: 56819, https://doi.org/10.53347/rID-56819
160	High-altitude pulmonary oedema	Case courtesy of Franco Ruales, Radiopaedia.org, rID: 16070, https://doi.org/10.53347/rID-16070
161	Hirschsprung disease	Case courtesy of Tee Yu Jin, Radiopaedia.org, rID: 71552, https://doi.org/10.53347/rID-71552
162	Hirschsprung disease	Case courtesy of Craig Hacking, Radiopaedia.org, rID: 73066, https://doi.org/10.53347/rID-73066
163	Horseshoe adrenal gland	Case courtesy of Caitriona Logan, Radiopaedia.org, rID: 78829, https://doi.org/10.53347/rID-78829
164	Horseshoe kidney and obstructive ureteral stone	Case courtesy of Vitalii Rogalskyi, Radiopaedia.org, rID: 66217, https://doi.org/10.53347/rID-66217
165	Horseshoe lung	Case courtesy of Rachael O'Rourke, Radiopaedia.org, rID: 38005, https://doi.org/10.53347/rID-38005
166	Huntington disease	Case courtesy of Frank Gaillard, Radiopaedia.org, rID: 29262, https://doi.org/10.53347/rID-29262
167	Huntington disease	Case courtesy of Pierre Wibawa, Radiopaedia.org, rID: 56139, https://doi.org/10.53347/rID-56139
168	Huntington disease	Case courtesy of Frank Gaillard, Radiopaedia.org, rID: 41811, https://doi.org/10.53347/rID-41811
169	Hydatid cyst - lung	Case courtesy of Mohammad Taghi Niknejad, Radiopaedia.org, rID: 20982, https://doi.org/10.53347/rID-20982
170	Hydatid cysts of the brain	Case courtesy of Roberto Schubert, Radiopaedia.org, rID: 19182, https://doi.org/10.53347/rID-19182
171	Hydropneumothorax	Case courtesy of Ian Bickle, Radiopaedia.org, rID: 87417, https://doi.org/10.53347/rID-87417
172	Hydropneumothorax	Case courtesy of Mohammad Taghi Niknejad, Radiopaedia.org, rID: 97603, https://doi.org/10.53347/rID-97603
173	Hydropneumothorax	Case courtesy of Prashant Mudgal, Radiopaedia.org, rID: 26675, https://doi.org/10.53347/rID-26675
174	Hydropneumothorax, pneumomediastinum, pneumoperitoneum and pneumoretroperitoneum	Case courtesy of Pir Abdul Ahad Aziz Qureshi, Radiopaedia.org, rID: 68101, https://doi.org/10.53347/rID-68101
175	Idiopathic scoliosis	Case courtesy of Frank Gaillard, Radiopaedia.org, rID: 3986, https://doi.org/10.53347/rID-3986
176	Ileal atresia	Case courtesy of Paresh K Desai, Radiopaedia.org, rID: 5871, https://doi.org/10.53347/rID-5871
177	Iliopsoas bursitis	Case courtesy of Nolan Walker, Radiopaedia.org, rID: 43169, https://doi.org/10.53347/rID-43169
178	Imperforate hymen	Case courtesy of Ali Alsmair, Radiopaedia.org, rID: 88653, https://doi.org/10.53347/rID-88653
179	Incarcerated femoral hernia with small bowel obstruction	Case courtesy of Chris O'Donnell, Radiopaedia.org, rID: 17037, https://doi.org/10.53347/rID-17037
180	Incisional hernia	Case courtesy of Chris O'Donnell, Radiopaedia.org, rID: 29584, https://doi.org/10.53347/rID-29584
181	Incisional hernia	Case courtesy of Frank Gaillard, Radiopaedia.org, rID: 35980, https://doi.org/10.53347/rID-35980
182	Incomplete miscarriage	Case courtesy of G Balachandran, Radiopaedia.org, rID: 5584, https://doi.org/10.53347/rID-5584
183	Indirect inguinal hernia	Case courtesy of Lam Van Le, Radiopaedia.org, rID: 198812, https://doi.org/10.53347/rID-198812
184	Infantile hepatic haemangioma	Case courtesy of Rade Kovač, Radiopaedia.org, rID: 70210, https://doi.org/10.53347/rID-70210
185	Infectious mononucleosis	Case courtesy of Yaïr Glick, Radiopaedia.org, rID: 91833, https://doi.org/10.53347/rID-91833
186	Inferior vena cava thrombosis	Case courtesy of Chris O'Donnell, Radiopaedia.org, rID: 46784, https://doi.org/10.53347/rID-46784
187	Inflammatory aortic aneurysm	Case courtesy of Ian Bickle, Radiopaedia.org, rID: 81197, https://doi.org/10.53347/rID-81197
188	Inflammatory arthritis	Case courtesy of Chris O'Donnell, Radiopaedia.org, rID: 16569, https://doi.org/10.53347/rID-16569
189	Inflammatory hepatic adenoma	Case courtesy of Abraão Kupske, Radiopaedia.org, rID: 43106, https://doi.org/10.53347/rID-43106
190	Infraspinatus tendon cleavage tear	Case courtesy of Yahya Baba, Radiopaedia.org, rID: 157891, https://doi.org/10.53347/rID-157891
191	Inguinal hernia	Case courtesy of Frank Gaillard, Radiopaedia.org, rID: 2623, https://doi.org/10.53347/rID-2623
192	Internal carotid aneurysm	Case courtesy of Frank Gaillard, Radiopaedia.org, rID: 4941, https://doi.org/10.53347/rID-4941
193	Interstitial lung disease	Case courtesy of Prashant Mudgal, Radiopaedia.org, rID: 40532, https://doi.org/10.53347/rID-40532
194	Intracerebral haemorrhage	Case courtesy of Prashant Mudgal, Radiopaedia.org, rID: 26131, https://doi.org/10.53347/rID-26131
195	Intracerebral haemorrhage	Case courtesy of Jeremy Jones, Radiopaedia.org, rID: 6153, https://doi.org/10.53347/rID-6153
196	Intracranial teratoma - suprasellar	Case courtesy of Frank Gaillard, Radiopaedia.org, rID: 17613, https://doi.org/10.53347/rID-17613
197	Intradural extramedullary schwannoma	Case courtesy of Varun Babu, Radiopaedia.org, rID: 48753, https://doi.org/10.53347/rID-48753
198	Intraparenchymal and intraventricular haemorrhage	Case courtesy of Hidayatullah Hamidi, Radiopaedia.org, rID: 55360, https://doi.org/10.53347/rID-55360
199	Intrathecal disc extrusion	Case courtesy of Frank Gaillard, Radiopaedia.org, rID: 68847, https://doi.org/10.53347/rID-68847
200	Intraventricular meningioma	Case courtesy of Frank Gaillard, Radiopaedia.org, rID: 92453, https://doi.org/10.53347/rID-92453
201	Ischaemic colitis	Case courtesy of Frank Gaillard, Radiopaedia.org, rID: 8300, https://doi.org/10.53347/rID-8300
202	Inferior vena cava (IVC) sarcoma	Case courtesy of Frank Gaillard, Radiopaedia.org, rID: 10158, https://doi.org/10.53347/rID-10158
203	Jejunal atresia	Case courtesy of Hisham Alwakkaa, Radiopaedia.org, rID: 56231, https://doi.org/10.53347/rID-56231
204	Kaposi sarcoma	Case courtesy of Stefan Tigges, Radiopaedia.org, rID: 94681, https://doi.org/10.53347/rID-94681
205	Kaposi sarcoma	Case courtesy of Ian Bickle, Radiopaedia.org, rID: 49628, https://doi.org/10.53347/rID-49628
206	Knee osteoarthritis with intra-articular body	Case courtesy of Henry Knipe, Radiopaedia.org, rID: 67340, https://doi.org/10.53347/rID-67340
207	Krukenberg tumour	Case courtesy of Mohammad Niknejad, Radiopaedia.org, rID: 63068, https://doi.org/10.53347/rID-63068
208	Large anterior diaphragmatic hernia	Case courtesy of Aneta Kecler-Pietrzyk, Radiopaedia.org, rID: 53257, https://doi.org/10.53347/rID-53257
209	Large bowel obstruction	Case courtesy of Ian Bickle, Radiopaedia.org, rID: 50391, https://doi.org/10.53347/rID-50391
210	Large bowel obstruction - colorectal carcinoma	Case courtesy of Varun Babu, Radiopaedia.org, rID: 18015, https://doi.org/10.53347/rID-18015
211	Large diaphragmatic hernia	Case courtesy of Mohammad Taghi Niknejad, Radiopaedia.org, rID: 84924, https://doi.org/10.53347/rID-84924
212	Large esophageal cancer with achalasia	Case courtesy of Mohammad Niknejad, Radiopaedia.org, rID: 89641, https://doi.org/10.53347/rID-89641
213	Large pancreatic pseudocyst with mass effect	Case courtesy of Craig Hacking, Radiopaedia.org, rID: 75918, https://doi.org/10.53347/rID-75918
214	Large pleural effusion	Case courtesy of Mark Holland, Radiopaedia.org, rID: 22691, https://doi.org/10.53347/rID-22691
215	Left atrial appendage aneurysm	Case courtesy of Josiah J. Marasigan, Radiopaedia.org, rID: 155824, https://doi.org/10.53347/rID-155824
216	Left lower lobe collapse	Case courtesy of Michael Lousick, Radiopaedia.org, rID: 70484, https://doi.org/10.53347/rID-70484
217	Left middle cerebral artery infarct	Case courtesy of David Cuete, Radiopaedia.org, rID: 35732, https://doi.org/10.53347/rID-35732
218	Left ventricular pseudoaneurysm	Case courtesy of Azza Elgendy, Radiopaedia.org, rID: 49266, https://doi.org/10.53347/rID-49266
219	Lobar haemorrhage	Case courtesy of Frank Gaillard, Radiopaedia.org, rID: 10678, https://doi.org/10.53347/rID-10678
220	Lobar intracerebral haemorrhage	Case courtesy of Mark Rodrigues, Radiopaedia.org, rID: 58781, https://doi.org/10.53347/rID-58781
221	Lobar intracerebral haemorrhage	Case courtesy of Mark Rodrigues, Radiopaedia.org, rID: 58531, https://doi.org/10.53347/rID-58531
222	Lumbar disc extrusion	Case courtesy of Mostafa Elfeky, Radiopaedia.org, rID: 76717, https://doi.org/10.53347/rID-76717
223	Lumbar disc herniation and spinal canal stenosis	Case courtesy of Bálint Botz, Radiopaedia.org, rID: 87940, https://doi.org/10.53347/rID-87940
224	Lumbar spinal canal stenosis	Case courtesy of Bruno Di Muzio, Radiopaedia.org, rID: 40590, https://doi.org/10.53347/rID-40590
225	Malignant gastric outlet obstruction	Case courtesy of Chris O'Donnell, Radiopaedia.org, rID: 21425, https://doi.org/10.53347/rID-21425
226	Medullary nephrocalcinosis	Case courtesy of Vikas Shah, Radiopaedia.org, rID: 164910, https://doi.org/10.53347/rID-164910
227	Medulloblastoma	Case courtesy of Frank Gaillard, Radiopaedia.org, rID: 5300, https://doi.org/10.53347/rID-5300
228	Medulloblastoma	Case courtesy of Hani M. Al Salam, Radiopaedia.org, rID: 10374, https://doi.org/10.53347/rID-10374
229	Medulloblastoma	Case courtesy of Hani M. Al Salam, Radiopaedia.org, rID: 11004, https://doi.org/10.53347/rID-11004
230	Medulloblastoma	Case courtesy of The Radswiki, Radiopaedia.org, rID: 11616, https://doi.org/10.53347/rID-11616
231	Meningioma	Case courtesy of Frank Gaillard, Radiopaedia.org, rID: 14109, https://doi.org/10.53347/rID-14109
232	Mesenteric ischaemia	Case courtesy of Mohammad Niknejad, Radiopaedia.org, rID: 89477, https://doi.org/10.53347/rID-89477
233	Metastatic insulinoma	Case courtesy of Maxime St-Amant, Radiopaedia.org, rID: 19080, https://doi.org/10.53347/rID-19080
234	Miliary tuberculosis	Case courtesy of Sakher Alkhaderi, Radiopaedia.org, rID: 40957, https://doi.org/10.53347/rID-40957
235	Miliary tuberculosis	Case courtesy of Zach Drew, Radiopaedia.org, rID: 75157, https://doi.org/10.53347/rID-75157
236	Mucinous adenocarcinoma of the prostate	Case courtesy of Albina Polianskaia, Radiopaedia.org, rID: 189269, https://doi.org/10.53347/rID-189269
237	Multicystic peritoneal mesothelioma	Case courtesy of Anastasia Belozertseva, Radiopaedia.org, rID: 60261, https://doi.org/10.53347/rID-60261
238	Multifocal round pneumonia	Case courtesy of Ian Bickle, Radiopaedia.org, rID: 77355, https://doi.org/10.53347/rID-77355
239	Multinodular lung adenocarcinoma	Case courtesy of Adrià Roset Altadill, Radiopaedia.org, rID: 96008, https://doi.org/10.53347/rID-96008
240	Multiple exostosis	Case courtesy of Maulik S Patel, Radiopaedia.org, rID: 9561, https://doi.org/10.53347/rID-9561
241	Multiple myeloma	Case courtesy of Mohammad Niknejad, Radiopaedia.org, rID: 61320, https://doi.org/10.53347/rID-61320
242	Multiple myeloma	Case courtesy of Ian Bickle, Radiopaedia.org, rID: 75534, https://doi.org/10.53347/rID-75534
243	Multiple sclerosis	Case courtesy of Bruno Di Muzio, Radiopaedia.org, rID: 14607, https://doi.org/10.53347/rID-14607
244	Myxopapillary ependymoma	Case courtesy of Frank Gaillard, Radiopaedia.org, rID: 5514, https://doi.org/10.53347/rID-5514
245	Necrotising enterocolitis	Case courtesy of Frank Gaillard, Radiopaedia.org, rID: 5961, https://doi.org/10.53347/rID-5961
246	Necrotising pancreatitis	Case courtesy of Saeed Soltany Hosn, Radiopaedia.org, rID: 20595, https://doi.org/10.53347/rID-20595
247	Necrotising pancreatitis	Case courtesy of Roberto Schubert, Radiopaedia.org, rID: 14470, https://doi.org/10.53347/rID-14470
248	Necrotizing enterocolitis	Case courtesy of Frank Gaillard, Radiopaedia.org, rID: 6355, https://doi.org/10.53347/rID-6355
249	Neurotoxoplasmosis	Case courtesy of Shu Su, Radiopaedia.org, rID: 76297, https://doi.org/10.53347/rID-76297
250	Normal abdominal radiograph	Case courtesy of Craig Hacking, Radiopaedia.org, rID: 40900, https://doi.org/10.53347/rID-40900
251	Normal abdominal radiograph	Case courtesy of Jeremy Jones, Radiopaedia.org, rID: 34067, https://doi.org/10.53347/rID-34067
252	Normal abdominal radiograph - paediatric	Case courtesy of Ian Bickle, Radiopaedia.org, rID: 46488, https://doi.org/10.53347/rID-46488
253	Normal acromioclavicular joint	Case courtesy of Frank Gaillard, Radiopaedia.org, rID: 36288, https://doi.org/10.53347/rID-36288
254	Nutmeg liver	Case courtesy of Bruno Di Muzio, Radiopaedia.org, rID: 49958, https://doi.org/10.53347/rID-49958
255	Nutmeg liver	Case courtesy of Michael P. Hartung, Radiopaedia.org, rID: 63845, https://doi.org/10.53347/rID-63845
256	Nutmeg liver	Case courtesy of Donna D'Souza, Radiopaedia.org, rID: 36058, https://doi.org/10.53347/rID-36058
257	Oesophageal cancer	Case courtesy of Ian Bickle, Radiopaedia.org, rID: 92065, https://doi.org/10.53347/rID-92065
258	Oesophageal web	Case courtesy of Henry Knipe, Radiopaedia.org, rID: 31198, https://doi.org/10.53347/rID-31198
259	Oligoastrocytoma	Case courtesy of Frank Gaillard, Radiopaedia.org, rID: 2637, https://doi.org/10.53347/rID-2637
260	Osteochondral fracture	Case courtesy of Ian Bickle, Radiopaedia.org, rID: 38164, https://doi.org/10.53347/rID-38164
261	Osteochondral injury with detached fragment	Case courtesy of Magdalena Chmiel-Nowak, Radiopaedia.org, rID: 76267, https://doi.org/10.53347/rID-76267
262	Osteochondroma	Case courtesy of Aditya Shetty, Radiopaedia.org, rID: 27292, https://doi.org/10.53347/rID-27292
263	Ovarian fibroma	Case courtesy of Ahmed Abdrabou, Radiopaedia.org, rID: 37611, https://doi.org/10.53347/rID-37611
264	Ovarian fibroma	Case courtesy of Roberto Schubert, Radiopaedia.org, rID: 14171, https://doi.org/10.53347/rID-14171
265	Ovarian torsion	Case courtesy of Ali Abougazia, Radiopaedia.org, rID: 23154, https://doi.org/10.53347/rID-23154
266	Ovarian vein thrombosis	Case courtesy of Hani M. Al Salam, Radiopaedia.org, rID: 9435, https://doi.org/10.53347/rID-9435
267	Pancoast tumour	Case courtesy of Craig Hacking, Radiopaedia.org, rID: 73294, https://doi.org/10.53347/rID-73294
268	Pancreatic pseudocyst	Case courtesy of Prashant Mudgal, Radiopaedia.org, rID: 30711, https://doi.org/10.53347/rID-30711
269	Pancreatic pseudocyst causing gastric outlet obstruction	Case courtesy of Chris O'Donnell, Radiopaedia.org, rID: 27728, https://doi.org/10.53347/rID-27728
270	Perforated diverticulitis with pneumoperitoneum and pneumoretroperitoneum	Case courtesy of Vikas Shah, Radiopaedia.org, rID: 88673, https://doi.org/10.53347/rID-88673
271	Perforated duodenal diverticulum	Case courtesy of Craig Hacking, Radiopaedia.org, rID: 42002, https://doi.org/10.53347/rID-42002
272	Perforated duodenal ulcer	Case courtesy of Mohamed Saber, Radiopaedia.org, rID: 86855, https://doi.org/10.53347/rID-86855
273	Perforated duodenal ulcer	Case courtesy of Mahmoud Yacout Alabd, Radiopaedia.org, rID: 42078, https://doi.org/10.53347/rID-42078
274	Perforated duodenal ulcer	Case courtesy of Liz Silverstone, Radiopaedia.org, rID: 171648, https://doi.org/10.53347/rID-171648
275	Perforated necrotising enterocolitis	Case courtesy of Jeremy Jones, Radiopaedia.org, rID: 62793, https://doi.org/10.53347/rID-62793
276	Perianal abscess	Case courtesy of Prashant Mudgal, Radiopaedia.org, rID: 39783, https://doi.org/10.53347/rID-39783
277	Perianal abscess	Case courtesy of Roberto Schubert, Radiopaedia.org, rID: 13758, https://doi.org/10.53347/rID-13758
278	Pericardial calcification	Case courtesy of Ahmed Abdrabou, Radiopaedia.org, rID: 27254, https://doi.org/10.53347/rID-27254
279	Pericardial calcification	Case courtesy of James Harvey, Radiopaedia.org, rID: 79051, https://doi.org/10.53347/rID-79051
280	Pineal cyst	Case courtesy of Frank Gaillard, Radiopaedia.org, rID: 9326, https://doi.org/10.53347/rID-9326
281	Pineal cyst	Case courtesy of Frank Gaillard, Radiopaedia.org, rID: 22772, https://doi.org/10.53347/rID-22772
282	Pineal germinoma	Case courtesy of Henry Knipe, Radiopaedia.org, rID: 180008, https://doi.org/10.53347/rID-180008
283	Pineocytoma causing hydrocephalus	Case courtesy of Frank Gaillard, Radiopaedia.org, rID: 5296, https://doi.org/10.53347/rID-5296
284	Pituitary macroadenoma with cavernous sinus invasion	Case courtesy of Frank Gaillard, Radiopaedia.org, rID: 5277, https://doi.org/10.53347/rID-5277
285	Pleural effusion	Case courtesy of Ian Bickle, Radiopaedia.org, rID: 26730, https://doi.org/10.53347/rID-26730
286	Pneumoperitoneum	Case courtesy of Hidayatullah Hamidi, Radiopaedia.org, rID: 60388, https://doi.org/10.53347/rID-60388
287	Pneumoperitoneum	Case courtesy of Anil Kumar Geetha Virupakshappa, Radiopaedia.org, rID: 198858, https://doi.org/10.53347/rID-198858
288	Primary central nervous system (CNS) lymphoma	Case courtesy of Frank Gaillard, Radiopaedia.org, rID: 5374, https://doi.org/10.53347/rID-5374
289	Primary colonic lymphoma	Case courtesy of Yaïr Glick, Radiopaedia.org, rID: 86700, https://doi.org/10.53347/rID-86700
290	Progressive multifocal leukoencephalopathy	Case courtesy of Ashesh Ishwarlal Ranchod, Radiopaedia.org, rID: 165786, https://doi.org/10.53347/rID-165786
291	Prostate cancer	Case courtesy of Henry Knipe, Radiopaedia.org, rID: 189782, https://doi.org/10.53347/rID-189782
292	Prostatomegaly	Case courtesy of The Radswiki, Radiopaedia.org, rID: 11820, https://doi.org/10.53347/rID-11820
293	Pulmonary alveolar microlithiasis	Case courtesy of Ahmed Abdrabou, Radiopaedia.org, rID: 51115, https://doi.org/10.53347/rID-51115
294	Pulmonary alveolar microlithiasis	Case courtesy of Sajoscha A. Sorrentino, Radiopaedia.org, rID: 14876, https://doi.org/10.53347/rID-14876
295	Pulmonary artery aneurysms	Case courtesy of Ahmed Abdrabou, Radiopaedia.org, rID: 32749, https://doi.org/10.53347/rID-32749
296	Pulmonary cryptococcosis	Case courtesy of Yune Kwong, Radiopaedia.org, rID: 32973, https://doi.org/10.53347/rID-32973
297	Pulmonary haemorrhage	Case courtesy of Oliver Hennessy, Radiopaedia.org, rID: 33691, https://doi.org/10.53347/rID-33691
298	Pulmonary hamartoma	Case courtesy of Yi-Jin Kuok, Radiopaedia.org, rID: 17407, https://doi.org/10.53347/rID-17407
299	Pulmonary neuroendocrine tumour	Case courtesy of Ian Bickle, Radiopaedia.org, rID: 36527, https://doi.org/10.53347/rID-36527
300	Pulmonary oedema	Case courtesy of Ian Bickle, Radiopaedia.org, rID: 88933, https://doi.org/10.53347/rID-88933
301	Pyogenic liver abscess	Case courtesy of Laughlin Dawes, Radiopaedia.org, rID: 35962, https://doi.org/10.53347/rID-35962
302	Pyopneumothorax	Case courtesy of Abdallah Al Khateeb, Radiopaedia.org, rID: 44606, https://doi.org/10.53347/rID-44606
303	Pyopneumothorax	Case courtesy of Lam Van Le, Radiopaedia.org, rID: 199927, https://doi.org/10.53347/rID-199927
304	Rectal diverticulum	Case courtesy of Esteban Calleja Montealegre, Radiopaedia.org, rID: 64583, https://doi.org/10.53347/rID-64583
305	Rectal mantle cell lymphoma	Case courtesy of Bruno Di Muzio, Radiopaedia.org, rID: 73148, https://doi.org/10.53347/rID-73148
306	Renal abscess and renal vein thrombosis	Case courtesy of Natalie Yang, Radiopaedia.org, rID: 9960, https://doi.org/10.53347/rID-9960
307	Renal artery stenosis	Case courtesy of Alexandra Stanislavsky, Radiopaedia.org, rID: 12313, https://doi.org/10.53347/rID-12313
308	Renal cell carcinoma	Case courtesy of Natalie Yang, Radiopaedia.org, rID: 9889, https://doi.org/10.53347/rID-9889
309	Renal rhabdoid tumour	Case courtesy of Jeremy Jones, Radiopaedia.org, rID: 24185, https://doi.org/10.53347/rID-24185
310	Rhabdomyolysis	Case courtesy of Craig Hacking, Radiopaedia.org, rID: 37698, https://doi.org/10.53347/rID-37698
311	Rheumatoid arthritis	Case courtesy of Roberto Schubert, Radiopaedia.org, rID: 16746, https://doi.org/10.53347/rID-16746
312	Rheumatoid arthritis	Case courtesy of Mohammad Niknejad, Radiopaedia.org, rID: 160684, https://doi.org/10.53347/rID-160684
313	Rheumatoid arthritis	Case courtesy of Frank Gaillard, Radiopaedia.org, rID: 2741, https://doi.org/10.53347/rID-2741
314	Rheumatoid arthritis	Case courtesy of Frank Gaillard, Radiopaedia.org, rID: 7245, https://doi.org/10.53347/rID-7245
315	Rheumatoid arthritis	Case courtesy of Samir Benoudina, Radiopaedia.org, rID: 74487, https://doi.org/10.53347/rID-74487
316	Right lower lobe collapse and consolidation	Case courtesy of Hani M. Al Salam, Radiopaedia.org, rID: 13284, https://doi.org/10.53347/rID-13284
317	Right upper lobe collapse	Case courtesy of Frank Gaillard, Radiopaedia.org, rID: 10552, https://doi.org/10.53347/rID-10552
318	Right upper lobe collapse	Case courtesy of Ian Bickle, Radiopaedia.org, rID: 46178, https://doi.org/10.53347/rID-46178
319	Right ventricular thrombus	Case courtesy of Frank Gaillard, Radiopaedia.org, rID: 23616, https://doi.org/10.53347/rID-23616
320	Ring-enhancing cerebral metastasis	Case courtesy of Frank Gaillard, Radiopaedia.org, rID: 5563, https://doi.org/10.53347/rID-5563
321	Round pneumonia	Case courtesy of Jeremy Jones, Radiopaedia.org, rID: 20012, https://doi.org/10.53347/rID-20012
322	Ruptured abdominal aortic aneurysm	Case courtesy of Cláudio Souza, Radiopaedia.org, rID: 13753, https://doi.org/10.53347/rID-13753
323	Ruptured abdominal aortic aneurysm	Case courtesy of Donna D'Souza, Radiopaedia.org, rID: 3828, https://doi.org/10.53347/rID-3828
324	Ruptured renal hydatid cyst	Case courtesy of Mohammad Taghi Niknejad, Radiopaedia.org, rID: 21349, https://doi.org/10.53347/rID-21349
325	Rupturing abdominal aortic aneurysm	Case courtesy of Michael P. Hartung, Radiopaedia.org, rID: 73709, https://doi.org/10.53347/rID-73709
326	Sarcoidosis	Case courtesy of Craig Hacking, Radiopaedia.org, rID: 71482, https://doi.org/10.53347/rID-71482
327	Sarcoidosis	Case courtesy of Frank Gaillard, Radiopaedia.org, rID: 6546, https://doi.org/10.53347/rID-6546
328	Sarcoidosis	Case courtesy of Dr Vardan Abrahamyan, Radiopaedia.org, rID: 95888, https://doi.org/10.53347/rID-95888
329	Sarcoidosis	Case courtesy of Abdallah Alqudah, Radiopaedia.org, rID: 50738, https://doi.org/10.53347/rID-50738
330	Sarcomatoid mesothelioma	Case courtesy of Phillippa Gray, Radiopaedia.org, rID: 59661, https://doi.org/10.53347/rID-59661
331	Scheuermann disease	Case courtesy of Henry Knipe, Radiopaedia.org, rID: 38701, https://doi.org/10.53347/rID-38701
332	Sinding-Larsen-Johansson disease	Case courtesy of Javier Fager, Radiopaedia.org, rID: 38972, https://doi.org/10.53347/rID-38972
333	Small bowel obstruction	Case courtesy of Ahmed Abdrabou, Radiopaedia.org, rID: 35721, https://doi.org/10.53347/rID-35721
334	Small bowel obstruction	Case courtesy of Ian Bickle, Radiopaedia.org, rID: 34633, https://doi.org/10.53347/rID-34633
335	Small bowel obstruction	Case courtesy of Aditya Shetty, Radiopaedia.org, rID: 28737, https://doi.org/10.53347/rID-28737
336	Small bowel obstruction due to left inguinal hernia	Case courtesy of Vikas Shah, Radiopaedia.org, rID: 74732, https://doi.org/10.53347/rID-74732
337	Small cell carcinoma of the bladder	Case courtesy of Matt A. Morgan, Radiopaedia.org, rID: 41619, https://doi.org/10.53347/rID-41619
338	Small cell lung cancer	Case courtesy of Royal Melbourne Hospital Respiratory, Radiopaedia.org, rID: 21890, https://doi.org/10.53347/rID-21890
339	Small cell lung cancer	Case courtesy of Natalie Yang, Radiopaedia.org, rID: 8601, https://doi.org/10.53347/rID-8601
340	Small cell lung cancer with left main pulmonary arterial thrombosis	Case courtesy of Mohammad Niknejad, Radiopaedia.org, rID: 21037, https://doi.org/10.53347/rID-21037
341	Superior mesenteric vein (SMV) thrombosis with long-segment jejunal infarction	Case courtesy of Michael P. Hartung, Radiopaedia.org, rID: 65365, https://doi.org/10.53347/rID-65365
342	Spinal cord metastasis	Case courtesy of Frank Gaillard, Radiopaedia.org, rID: 9771, https://doi.org/10.53347/rID-9771
343	Splenic and hepatic tuberculous granulomatosis	Case courtesy of Michael P. Hartung, Radiopaedia.org, rID: 70558, https://doi.org/10.53347/rID-70558
344	Spondylolysis	Case courtesy of Abdel-Rahman Abdel-Halim, Radiopaedia.org, rID: 31063, https://doi.org/10.53347/rID-31063
345	Subarachnoid haemorrhage	Case courtesy of István Kui, Radiopaedia.org, rID: 83557, https://doi.org/10.53347/rID-83557
346	Subdural haemorrhage	Case courtesy of Frank Gaillard, Radiopaedia.org, rID: 35891, https://doi.org/10.53347/rID-35891
347	Subdural haemorrhage	Case courtesy of Frank Gaillard, Radiopaedia.org, rID: 17559, https://doi.org/10.53347/rID-17559
348	Subdural hematoma, uncal herniation and Duret brainstem hemorrhage	Case courtesy of Andrew Dixon, Radiopaedia.org, rID: 32383, https://doi.org/10.53347/rID-32383
349	Tension hemothorax	Case courtesy of Rania Adel Anan, Radiopaedia.org, rID: 157452, https://doi.org/10.53347/rID-157452
350	Terminal ileal tuberculosis	Case courtesy of Vikas Shah, Radiopaedia.org, rID: 68643, https://doi.org/10.53347/rID-68643
351	Tetralogy of fallot	Case courtesy of Frank Gaillard, Radiopaedia.org, rID: 8049, https://doi.org/10.53347/rID-8049
352	Tetralogy of fallot	Case courtesy of Vincent Tatco, Radiopaedia.org, rID: 43227, https://doi.org/10.53347/rID-43227
353	Thalamic glioma	Case courtesy of Bianca Larissa Pereira, Radiopaedia.org, rID: 66119, https://doi.org/10.53347/rID-66119
354	Thalamic lacunar infarct	Case courtesy of Roberto Schubert, Radiopaedia.org, rID: 14098, https://doi.org/10.53347/rID-14098
355	Thalamic lacunar infarct	Case courtesy of David Cuete, Radiopaedia.org, rID: 36507, https://doi.org/10.53347/rID-36507
356	Thalamic lacunar infarct	Case courtesy of Bahman Rasuli, Radiopaedia.org, rID: 98965, https://doi.org/10.53347/rID-98965
357	Thrombosed right and left coronary artery aneurysms	Case courtesy of Stefan Tigges, Radiopaedia.org, rID: 98013, https://doi.org/10.53347/rID-98013
358	Tonsillar abscess	Case courtesy of Frank Gaillard, Radiopaedia.org, rID: 4942, https://doi.org/10.53347/rID-4942
359	Transposition of the great arteries	Case courtesy of Vincent Tatco, Radiopaedia.org, rID: 43062, https://doi.org/10.53347/rID-43062
360	Traumatic central cord syndrome	Case courtesy of Arthur Daire, Radiopaedia.org, rID: 30935, https://doi.org/10.53347/rID-30935
361	Trichobezoar - Rapunzel syndrome	Case courtesy of Maryam Shekarforoush, Radiopaedia.org, rID: 150117, https://doi.org/10.53347/rID-150117
362	Trigeminal schwannoma	Case courtesy of Frank Gaillard, Radiopaedia.org, rID: 10433, https://doi.org/10.53347/rID-10433
363	Tuberculous autonephrectomy	Case courtesy of Natalie Yang, Radiopaedia.org, rID: 9933, https://doi.org/10.53347/rID-9933
364	Ulcerative colitis	Case courtesy of Mohammad Niknejad, Radiopaedia.org, rID: 64578, https://doi.org/10.53347/rID-64578
365	Ulcerative colitis	Case courtesy of Hala Maher, Radiopaedia.org, rID: 83013, https://doi.org/10.53347/rID-83013
366	Ulcerative colitis	Case courtesy of Andrew Dixon, Radiopaedia.org, rID: 28563, https://doi.org/10.53347/rID-28563
367	Urinary bladder calculus	Case courtesy of Aditya Shetty, Radiopaedia.org, rID: 27089, https://doi.org/10.53347/rID-27089
368	Urinary bladder calculus	Case courtesy of Jeremy Jones, Radiopaedia.org, rID: 6141, https://doi.org/10.53347/rID-6141
369	Urinary bladder calculus	Case courtesy of Hoe Han Guan, Radiopaedia.org, rID: 150346, https://doi.org/10.53347/rID-150346
370	Urinary bladder diverticulum	Case courtesy of Mohammad Taghi Niknejad, Radiopaedia.org, rID: 93155, https://doi.org/10.53347/rID-93155
371	Usual interstitial pneumonia	Case courtesy of Bruno Di Muzio, Radiopaedia.org, rID: 81350, https://doi.org/10.53347/rID-81350
372	Usual interstitial pneumonia	Case courtesy of David Cuete, Radiopaedia.org, rID: 27858, https://doi.org/10.53347/rID-27858
373	Uterus didelphys with hematometrocolpos	Case courtesy of Ahmed Abdrabou, Radiopaedia.org, rID: 35795, https://doi.org/10.53347/rID-35795
374	Vesicoureteric junction lithiasis	Case courtesy of David Puyó, Radiopaedia.org, rID: 18334, https://doi.org/10.53347/rID-18334
375	Xanthogranulomatous pyelonephritis with perinephric abscess	Case courtesy of Frank Gaillard, Radiopaedia.org, rID: 16883, https://doi.org/10.53347/rID-16883
376	Yolk sac tumour	Case courtesy of Levon Davtyan, Radiopaedia.org, rID: 153896, https://doi.org/10.53347/rID-153896
377	Zenker diverticulum	Case courtesy of Mohammad Taghi Niknejad, Radiopaedia.org, rID: 191511, https://doi.org/10.53347/rID-191511
